# Gender, Tobacco Control Policies, and Recalcitrant Smoking Among Older Adults in 20 European Countries

**DOI:** 10.1093/geroni/igab046.1913

**Published:** 2021-12-17

**Authors:** Manjing Gao, Chioun Lee, Soojin Park

**Affiliations:** University of California, Riverside, Riverside, California, United States

## Abstract

Little is known about sociodemographic and macro-level predictors of “recalcitrant smoking,” defined as persistent smoking when one has developed a health condition that is likely caused by smoking. We aim to investigate the impact of gender, education, and tobacco control policies on recalcitrant smoking among older adults in Europe from 2006 through 2013. Data from 33,839 respondents—aged 50 years and older with a smoking history and at least one smoking-related health condition—were pooled from the 2006–07, 2011, and 2013 waves of three harmonized longitudinal studies on ageing (SHARE, ELSA, and TILDA). We fitted gender-specific logistic regression models with two-way fixed effects and tested interaction terms between gender, tobacco control policies, and education, adjusting for age, marital status, GDP per capita, smoking prevalence, country, and year-fixed effects. Compared to men and individuals with higher levels of education, women and less educated individuals were more likely to be recalcitrant smokers. The association between education and recalcitrant smoking was stronger for women than men. The inverse association between the TCS and recalcitrant smoking was stronger for those having upper secondary education (for men: OR = 0.905, CI = 0.849–0.965; for women: OR = 0.897, CI = 0.834–0.964) and tertiary education (for men: OR = 0.802, CI = 0.717–0.898; for women: OR = 0.739, CI = 0.603–0.907), compared to those having less than upper secondary. As women and less educated individuals are vulnerable to recalcitrant smoking, future policies targeting these marginalized groups are needed to prevent recalcitrant smoking in old age.

